# Dielectric Properties of PEEK/PEI Blends as Substrate Material in High-Frequency Circuit Board Applications

**DOI:** 10.3390/mi15060801

**Published:** 2024-06-18

**Authors:** Tim Scherzer, Marius Wolf, Kai Werum, Holger Ruckdäschel, Wolfgang Eberhardt, André Zimmermann

**Affiliations:** 1 Department of Polymer Engineering, University of Bayreuth, Universitätsstrasse 30, 95447 Bayreuth, Germany; 2 Institute for Micro Integration (IFM), University of Stuttgart, Allmandring 9b, 70569 Stuttgart, Germany; 3 Hahn-Schickard, Allmandring 9b, 70569 Stuttgart, Germany

**Keywords:** dielectric properties, permittivity, dielectric loss factor, polymer blend, PEEK, PEI, high frequency, laser direct structuring, printed circuit board

## Abstract

Substrate materials for printed circuit boards must meet ever-increasing requirements to keep up with electronics technology development. Especially in the field of high-frequency applications such as radar and cellular broadcasting, low permittivity and the dielectric loss factor are key material parameters. In this work, the dielectric properties of a high-temperature, thermoplastic PEEK/PEI blend system are investigated at frequencies of 5 and 10 GHz under dried and ambient conditions. This material blend, modified with a suitable filler system, is capable of being used in the laser direct structuring (LDS) process. It is revealed that the degree of crystallinity of neat PEEK has a notable influence on the dielectric properties, as well as the PEEK phase structure in the blend system developed through annealing. This phenomenon can in turn be exploited to minimize permittivity values at 30 to 40 wt.-% PEI in the blend, even taking into account the water uptake present in thermoplastics. The dielectric loss follows a linear mixing rule over the blend range, which proved to be true also for PEEK/PEI LDS compounds.

## 1. Introduction

Communication electronics in industry and commercial use have to deal with ever-increasing signal data rates and frequencies. The latter allows applications in the fifth-generation standard for broadband cellular networks (5G), its successor 6G already being under development. Here, millimeter-wave technology (mmWave) plays a vital role as well as short-range directed radar technologies, e.g., essential for automated transportation. All those developments rely on signal frequencies in the GHz range: 5G being designed for the utilization of frequencies up to 71 GHz, mmWave using 30–300 GHz and current automotive radar technologies relying on frequencies of up to 77 GHz [1]. Antenna structures are mainly structured directly on the surface of the printed circuit board (PCB), allowing for higher system integrity. To achieve the latter, integration of conductive tracks into already highly integrated devices (MID: Molded Interconnect/Mechatronic Integrated Devices) via laser structuring and metallization is already established. First investigations have already been carried out to test the applicability of these materials for the high-frequency (HF) range [2]. However, with rising signal frequencies, the signal dampening properties of the PCB substrate material become more and more important, as shorter electromagnetic wave lengths in the relevant GHz regime become increasingly prone to absorption in matter [3,4]. In this case, dielectric properties of the substrate material, i.e., (relative) permittivity and loss factor, are of particular importance.

Permittivity, *ε*, describes the polarizability of a dielectric subjected to an external electromagnetic field. In practice, permittivity is often expressed as a relative permittivity, *ε_r_*, comparing a given dielectric medium to the absolute permittivity of a vacuum, *ε*_0_:

(1) $$\varepsilon =\varepsilon_{r}\varepsilon_{0}$$

In an alternating electric field, such as radio waves, permittivity becomes a complex value,

(2) $$\varepsilon_{r}^{*}=\varepsilon_{r}^{\prime }-i\varepsilon_{r}^{\prime \prime }$$

For the sake of readability, the subscript *r* is often neglected, as it will be in this work. Here, the real part, *ε*′, represents the stored energy within the dielectric subjected to an electric field, while the imaginary part, *ε*″, represents the dielectric losses resulting in an increase in material temperature as an observable macroscopic effect [5,6]. For characterization and comparison of dielectric properties in PCB applications, most commonly the parameters *ε*′ and tan*δ* of a given material are investigated, where the latter represents the ratio of the imaginary to real part of ε*,

(3) tanδ=ε″/ε′

Antenna and general PCB layout designs for high-frequency (HF) applications benefit from substrate materials with permittivity and loss factor values as low as possible. This fact is underpinned by a look at currently commercially available PCB substrate materials, as listed in Table 1. Permittivity values *ε*′ of 3.0 or below are desired, as well as a low loss factor tan*δ* in the per mille range or below.

As can be seen, all PCB base materials are either based on thermoset resins or fluorinated PTFE material. PTFE is widely seen as the gold standard for HF PCB materials, as it offers the lowest dielectric values for polymers (ε′ 2.1, tanδ 0.0002). The addition of various filler substances improves the mechanical behavior and aids matching the coefficient of thermal expansion of the polymer-based substrate with the applied copper to form conductive tracks on the PCB. Both substrate materials, however, come with drawbacks: Resin-based substrates can hardly be recycled on a material level and are mostly landfilled, burned or shredded and partly used as filler, e.g., in construction materials [7]. PTFE, on the other hand, poses possible health hazards, with its negative impact on human health through its longevity, which is still under investigation and not conclusively clarified [8]. Here, thermoplastics can offer a possible alternative substrate material base, as they are re-meltable and recycling routes on a material level are already established. To take into account the temperature demands on PCB substrate materials, i.e., thermal loads in soldering processes and possible heat built-up during the use phase, only high-temperature engineering thermoplastics can be considered here.

A PCB manufacturing process that makes use of the intrinsic re-meltable thermoplastic material property is the technology of laser direct structuring (LDS). A plethora of literature can be found elsewhere detailing this process, which comprises the following steps:

(I) Conventional injection molding of a thermoplastic part, using compounds containing the respective LDS additive; (II) Ablation of areas on the part’s surface to be metallized, i.e., locally removing the thermoplastic matrix using laser to expose and activate the neat LDS additive; (III) Accumulating copper/silver/etc. on the exposed areas via electroless metallization. Ref. [9] Several LDS-capable compounds are commercially available, offering a wide range of thermoplastic matrices. Here, for high-temperature demands during PCB processing, soldering steps in particular, engineering plastics are preferred.

For this reason, we selected blends of PEEK and PEI for investigations over the whole blend range in order to determine their potential use for the proposed application. Both materials offer the lowest dielectric properties in the field of engineering plastics, just at the very edge of HF capability, as provided by data sheets (PEI: *ε*′ 3.02, tan*δ* 0.0027, both @ 10 GHz; PEEK: *ε*′ 3.10 @ 1 kHz, tan*δ* 0.0040 @ 1 MHz) [10,11]. There are no studies in the available literature that take into account the influence of crystallinity when investigating the dielectric properties of PEEK in the GHz range. Reported dielectric values for PEI in the GHz range do scatter, yet are consistently lower than those of PEEK. The influence of humidity is rarely addressed. Table 2 shows representative reported ε′ and tanδ values for PEEK and PEI.

PEEK/PEI blend systems have been investigated quite thoroughly in the past, with first insights into their complete miscibility in the late 1980s [18,19,20,21]. The overarching findings related to this work are as follows: (I) The crystalline structure of the PEEK fraction in the blend remains mostly the same over the blend range through the exclusion of PEI from those structures during crystalline growth; (II) The overall crystallization kinetic is slowed down, caused by entanglement of amorphous PEI and the mobile amorphous fraction of PEEK in the melt state [20,22,23,24]. Utilizing this behavior facilitates the blend’s application for improved tribological and mechanical properties compared to the neat thermoplastics [25,26], as well as novel approaches for fused filament fabrication additive manufacturing [27] and blend structure exploitation for porous PEEK membranes [28], among others.

What is lacking in the literature, to date, is an investigation of the dielectric properties of this blend system for the proposed PCB substrate application, especially in the GHz frequency range. An influence of the blend ratio and crystalline content on permittivity and loss factor values is expected, but not yet proven. With respect to the LDS process, this becomes particularly interesting, as PEEK-based LDS compounds are available but not yet investigated in terms of crystallinity-influenced dielectric properties in the GHz range. The present work aims to close this gap in PEEK/PEI blend applicability for high-frequency environments. Furthermore, the findings are to be applied to a commercial PEEK/PEI-based compound used for said LDS processes, further proving the relevance of this investigation route even for filled thermoplastic compounds.

## 2. Materials and Methods

### 2.1. Materials

Common commercial PEEK and PEI grades were chosen in order to allow a comparison of results with those in the existing literature. Specifically, PEEK 450P (Victrex plc, Thornton Cleveleys, UK)—a powder variant of type 450G—and PEI Ultem 1000 (Sabic, Riyadh, Saudi Arabia) were used for the thermoplastic blends under investigation. For the transfer trials, the authors made use of the commercial compound Tecacomp PEEK LDS black 1,047,045 (Ensinger GmbH, Nufringen, Germany). For blending, a second compound was produced by Ensinger based on Ultem 1000 matrix, the filler being the same Tecacomp filler material and content, not to be disclosed here.

### 2.2. Processing

PEEK/PEI blends were produced in 100:0 … 0:100 ratios in 10 wt.-% increments using a micro-compounder MC 15 HT (Xplore, Sittard, The Netherlands). Nomenclature throughout this work will be as follows: PEIXX, where XX represents the wt.-% of PEI in the thermoplastic blend (e.g., PEI70 indicating a PEEK:PEI wt.-ratio of 30:70). Processing parameters were set to a temperature of 390 °C, a screw speed of 100 rpm and a mixing time of 3 min, with nitrogen purging the feed section of the device. Pre-trials showed stable processing parameters attributed to melt viscosity and machine torque after ca. 2 min, hence an additional minute of mixing time was added for good measure. As the device only allows for a batch-wise processing of nominal 15 cm³ material (melt volume), consecutive runs for the same blends were carried out. Taking into account material releasing and refilling, six consecutive runs led to an overall processing time of 25 to 28 min per blend. Neat materials, i.e., PEI0 (pure PEEK) and PEI100 (pure PEI) did not undergo a compounding run, as the authors considered the degrading impact of the compounder on PEEK and PEI to be negligible.

Specimen production and annealing were carried out using manual hot presses (Paul-Otto Weber GmbH, Remshalden, Germany). Granules cut from the extruded strands, as-received PEI granules (PEI100) and as-received PEEK powder (PEI0) were pressed into square plates using a rudimentary three-plate mold setup with an overall stack thickness of 2.5 mm. This process leads to a nominal specimen size of 51 × 51 × 0.5 mm^3^, yet through mold overfilling, the final parts showed thicknesses between 0.6 to 0.7 mm. Hot pressing was carried out at 390 °C with a pressing force of 25 kN, producing a pressure of roughly 100 bar on the projected mold area in order to simulate usual processing pressures in injection molding or extrusion. After preheating the material in the mold for 3 min with the hot plates in bare contact, pressure was applied for 1 min. Mold plates were then swiftly transferred to a water-cooled press subjected to the same pressure for 2 min, leading to an overall processing time of 6 min. To prevent material from sticking to the mold plates, they were pre-treated with a high-temperature release agent HT400+ (Haufler Composites GmbH&Co. KG, Blaubeuren, Germany). For quenching trials of neat PEEK, heating and pressing followed the same routine, while quenching was achieved by immersing the mold stack into a water bath kept at ambient temperature for faster heat dissipation.

Annealing was carried out with the same presses at 305 °C for 1 h. Specimens were placed between two pre-heated sheet-metal plates covered with polyimide release foil with the heated plates barely in contact to ensure heat transfer yet avoid excessive specimen deformation. After 1 h, the press temperature was set to 130 °C (below any blend’s T_g_), which took roughly 0.5 h to reach. Subsequently, the stack was cooled down to ambient temperature with a water-cooled press. Prior to any processing, all materials were dried overnight at 120 °C using a vacuum oven to remove moisture.

In terms of sample designation, the following nomenclature is used throughout the results and discussion: “as-processed” describing a specimen originating from the hot-pressing process described above via the cold-press cooling; “quenched” referring to a water-quenched specimen; “annealed” meaning a specimen that has undergone the outlined annealing routine, coming from either the as-processed or the quenched state.

### 2.3. Analytics

For crystallinity determination and crystallization kinetics investigation, differential scanning calorimetry (DSC) analyses were carried out using a DSC 1 (Mettler-Toledo AG, Schwerzenbach, Switzerland) under nitrogen atmosphere with sample weights between 5.0 and 6.0 mg in aluminum crucibles. If not stated otherwise, a heating/cooling rate of 10 K/min was applied with a 1 min isothermal between dynamic sections. The degree of crystallization of the semicrystalline PEEK in the blend Χ_c,PEEK_ was determined by calculating the enthalpy of fusion from the area under the endotherm melting peak via the Mettler-Toledo STARe software (version 18.00a) with a spline baseline applied. The commonly used calculation of Χ_c,PEEK_

(4) $${Χ}_{c,PEEK}={(\Delta H}_{m}^{\;}-{∆H}_{cc})/\left({\Delta H}_{m}^{0}\times w_{PEEK}\right)$$

was used, with ΔH_m_ being the determined enthalpy of fusion from the integrated DSC curve, ΔH_cc_ the enthalpy of potential cold crystallization appearing, ΔH_m_^0^ the enthalpy of fusion of the totally crystalline polymer, and w_PEEK_ the weight-fraction of PEEK in the PEEK/PEI blend system. Hereby, for neat PEEK, the value of ΔH_m_^0^ = 130 J/g used in the literature was assumed [29]. As Χ_c_ determination via DSC is prone to deviation by small changes in the setting of integration borders and baseline curvature, crystallinity values are given in whole % numbers. Additionally, DSC runs with cooling rates of −10/−20/−30 K/min were conducted to determine crystallization halftimes.

Auxiliary isothermal DSC runs performed at 305 °C from the quenched samples, mimicking the annealing process, revealed that even for the blend to be expected with the slowest crystallization speed, i.e., PEI90, no exothermic processes could be recognized after ca. 30 min isothermal. To account for processing uncertainties and ensure maximum crystallization throughout the entire sample range, an annealing time of 1 h was chosen for all blend ratios.

Modulated DSC (mDSC) was carried out using a Q1000 (TA Instruments, New Castle, DE, USA). Here, reversing and nonreversing heat flow signals could be split by applying a modulated temperature program. This allowed for the separation of overlapping thermal events, providing better insight into both kinetic and thermodynamic material properties. In order to detect even small reversing effects, material samples of 10.0 to 11.0 mg were treated with a 2 K/min heating rate with an overlaid modulation amplitude of 1.59 K/60 s period. For relaxation and T_g_ determination, the reversible heat capacity c_p_ signal was used for analysis with the TA Instruments Universal Analysis 2000 software (version 4.5A). Δc_p_ at T_g_ was calculated via the signal change between the two onset temperatures automatically detected through the T_g_ determination.

DMA trials for T_g_ determination were performed with an RDAIII (TA Instruments, New Castle, DE, USA). Specimens of 50 × 10 mm^2^ dimensions were punched from the pressed plates to be tested in torsion (1 Hz, 0.1% strain) at heating rates of 3 and 10 K/min in a range from 50 to 230 °C.

A P9374A vector network analyzer (Keysight, Santa Rosa, CA, USA) was used to determine the dielectric properties of the materials. Split-post dielectric resonators (QWED Sp. z o.o., Warsaw, Poland) at discrete resonating frequencies of 5 and 10 GHz, respectively, were used to measure complex permittivity values of *ε*′ and *ε*′′ as well as the loss factor tan*δ*.

Density measurements were carried out via hydrostatic weighing using an AG245 weighing scale (Mettler Toledo, Greifensee, Switzerland).

Material water uptake was calculated utilizing the full-immersion variant. The pressed specimens were dried as described above, weighed dry, completely immersed in DI water for 96 h and weighed again, assuming a close-to-equilibrium water uptake due to the low specimen thickness.

For data display, error bars represent the single standard deviation of measured values. Gaussian distribution of measured values was checked by employing a Shapiro–Wilk test, which, among other normality tests, delivers useful results for sample sizes < 50 [30,31]. However, the authors are aware of the limited validity of those results due to the rather small sample size.

## 3. Results and Discussion

### 3.1. Neat PEEK

In order to initially prove the presence of a crystallinity-dependent shift in the dielectric properties at the frequencies under investigation, neat PEEK was processed, quenched and annealed to obtain two morphologies with distinctively different degrees of crystallinity.

Since correct baseline placement through integration borders as well as type of baseline are highly subjective to the individual investigator, with additional issues of superimposed cold crystallization and melting effects possible [32,33], material annealing was utilized to produce sharp melting endotherm onsets in the DSC curve as well as to eliminate any exothermic recrystallization effects prior to endothermic melting, which oftentimes leads to an overestimation in PEEK crystallinity determination via DSC [34]. Annealing of PEEK leads to the formation of a second melting endotherm peak, an effect well-described in the literature [35]. For this study, an annealing temperature of 305 °C was chosen after consulting literature dealing with annealing of neat PEEK, PEEK blends and composites [18,19,27,36,37]: This annealing temperature maximizes the achievable relative PEEK crystallinity through all blend ratios and shifts the emerging second endothermic peak closer to the initial PEEK melting peak. Also, it delivers a sharp onset in the curve’s slope, hence reducing the influence of operator-dependent choice of baseline boundary position. At the same time, this temperature still allows for annealing of samples coming from the glassy state for all blend ratios without excessive specimen deformation, which from a part production/processing perspective, is closer to an actual application than coming from the molten state (e.g., for injection molding: post hoc part annealing after manufacturing instead of expanding cycle time due to timely extended annealing/cooling within the mold). It has been shown for a 300 °C annealing temperature that Χ_c,PEEK_ values are virtually on par for isothermal melt-crystallization compared to cold-crystallization from the glassy state [37].

**Figure 1:**
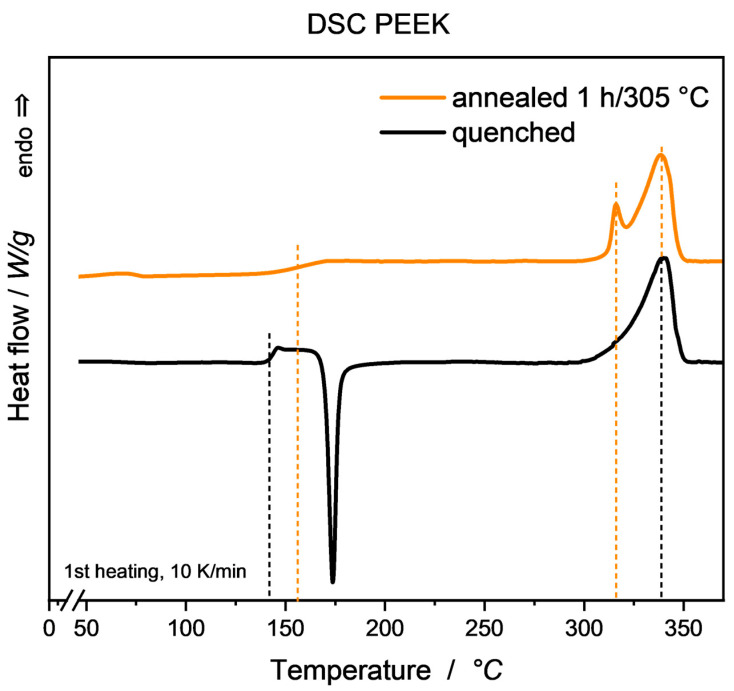
Exemplary DSC curves of quenched and annealed PEEK (1st heating). Through quenching, a strong exothermic recrystallization peak can be observed, while the annealed sample shows a rise in T_g_ as well as a characteristic second endothermic peak slightly above annealing temperature. Respective temperatures are indicated by dashed lines. Resulting measured degrees of crystallinity Χc are at 20% (quenched) and 37% (annealed), respectively.

**Figure 2:**
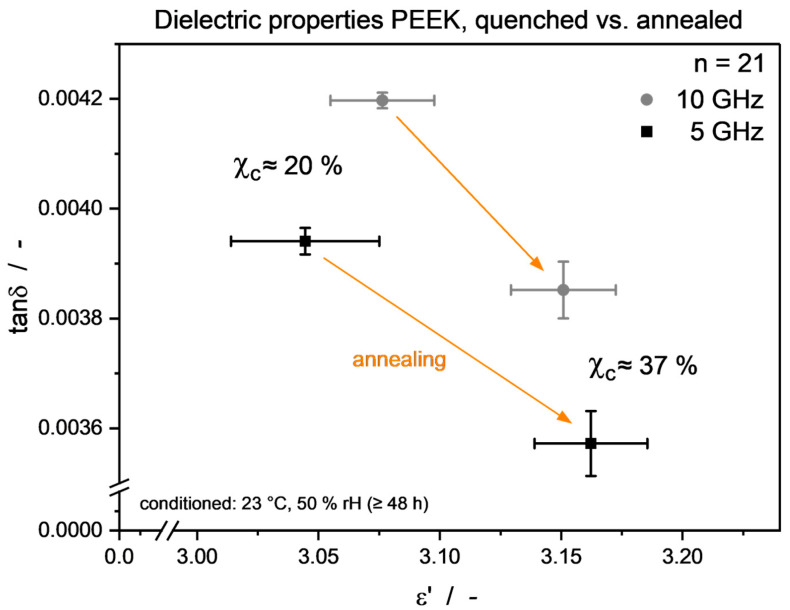
PEEK dielectric properties of quenched and annealed specimen. An apparent shift to higher ε′ as well as lower tanδ can be seen.

Figure 1 exemplarily depicts the 1st DSC heating curves for the quenched and annealed PEEK sample, respectively, highlighting the annealing effects mentioned and referenced above, i.e., formation of a second endothermic peak, sharper endothermic onset, as well as a shift to higher T_g_. The latter effect is evoked by the decreased immobilization of the amorphous fraction due to incorporation of molecules formerly attributed to the mobile amorphous phase into crystalline regions, thus requiring a higher energy for molecular motion, and is well-described throughout the literature [19]. Measurements of dielectric properties on quenched and annealed samples have been carried out with samples stored at ambient room conditions. In Figure 2, a visible trend towards higher permittivity *ε*′ and lower loss factor tan*δ* can be seen, caused by an increase in material crystallinity. Comparative literature on this effect, especially in the GHz regime, is quite sparse. However, from findings for different materials and frequency ranges, both effects can be explained to some extent.

The root cause of both effects can be found in the underlying structure–property relationships. Permittivity itself describes a volumetric material property. More precisely, the measured value is the relative permittivity, comparing a bulk material permittivity to that of vacuum (*ε*_0_) that is considered a physical constant. If the molecular free volume per given volume unit within a material increases, e.g., per selective atomic substitution, addition of bulky side groups in a polymer molecule, or deliberately micro-branched crosslinking [38,39,40], the measured permittivity of said material can be reduced. More applicable to our investigations, however, is a reduction in free volume through an increase in the degree of crystallinity, reflected by an increase in the material’s density. The literature shows this effect to be apparent within several polymeric materials [41,42], however, only in measuring frequencies below 1 GHz.

The dielectric losses, as denominated by *ε*″, can be attributed to molecular mobility. In the literature, the mobility of polymers has mainly been varied by changes in measuring temperature [43,44]. The influence of Χ_c_ on dielectric losses has also been reported [42]; however, interactions with crystallinity-affecting fillers have to be considered as well. It has been proposed that the molecular mobility of polymers can be increased in the filler particle vicinity, leading to the formation of an interfacial region with higher local free volume and mobility, and thus contributing to a higher *ε*″ [43]. All those findings can in turn be applied to the tan*δ* used as a dielectric loss descriptor in this work, as the measured Δ*ε*′ (i.e., difference in *ε*′ between quenched and annealed state) is much smaller than the respective *ε*′ measurement values. Hence, it can be seen as nearly constant in (3), leading to tan*δ*~*ε*″.

### 3.2. Blends

To go further into detail concerning the dielectric properties of PEEK/PEI blends and evaluate the pure material properties, the influence of moisture has to be removed from the system under investigation. As is known, water molecules exhibit a high dipole moment due to the difference in electronegativity of H and O atoms. Water shows a decreasing yet still high relative permittivity of 60–80 in the range of 1–10 GHz, as well as high loss factors from about 20 to 40, depending on the salinity [45,46]. Hence, it is to be expected that the presence of moisture in the thermoplastic material will mask the actual material properties, which are lower by orders of magnitude. To compare the impact of moisture, all dielectric measurements of the blended polymers were taken in a conditioned sample state (23 °C, 50% RH) by use of a climate chamber, as well as a dried state using the material drying process mentioned above.

**Figure 3:**
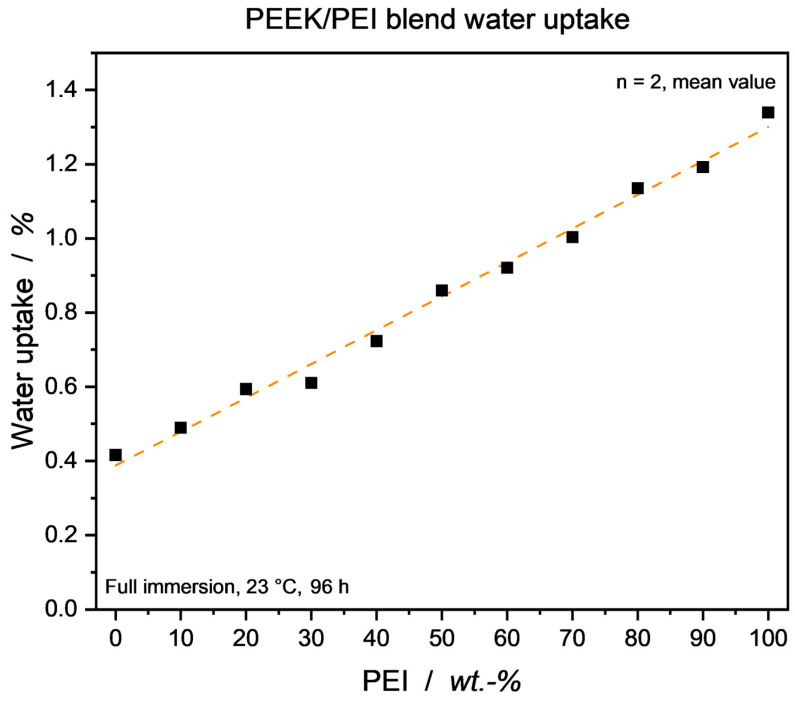
PEEK/PEI blend water uptake via full immersion method. Annealed samples were used. Linear interpolation to guide the eye.

**Figure 4:**
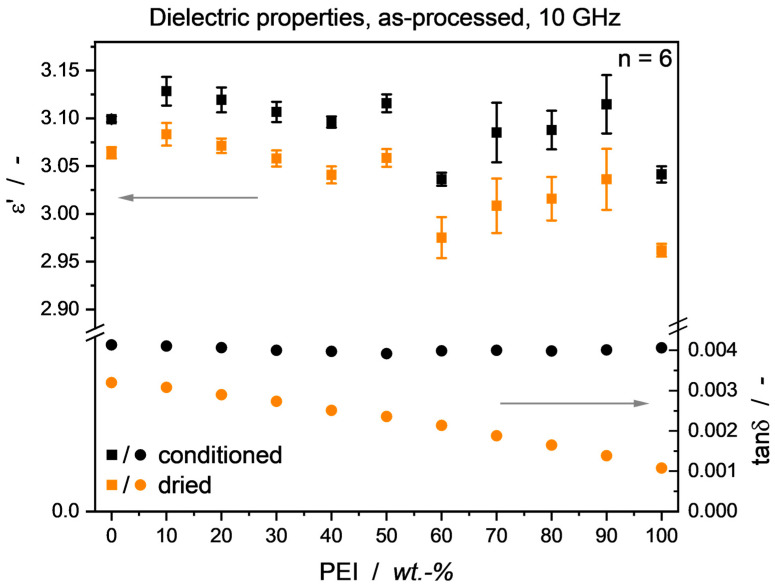
Impact of moisture on blend dielectric properties. For permittivity ε′, a linear shift to lower values can be seen after drying. The change in loss factor tanδ is heavily correlated with moisture content, effectively masking the actual material behavior. Note that tanδ standard deviation bars are hidden due to the diagram scale. Material samples were as-processed from the hot press.

Considering the neat PEEK and PEI, measured water uptake for the annealed specimen was in good accordance with the materials’ data sheets [10,11]. As seen in Figure 3, a close to linear behavior throughout the blend range can be observed. Without discussing water diffusion mechanisms in miscible thermoplastic blends in detail, a similar trend can be seen in other compatibilized blend systems [47], yet deviating behavior is also reported [48,49]. The influence of moisture in the material can be observed in Figure 4, as a clear trend towards lower dielectric values in the absence of water is visible. While the change in permittivity *ε*′ is only marginally dependent on the percentage of water, the loss factor tan*δ* exhibits a clear correlation with the amount of water present over the blend range, i.e., the higher water uptake of the PEI-rich blends also leads to a larger decrease in tan*δ* values through drying.

**Figure 5:**
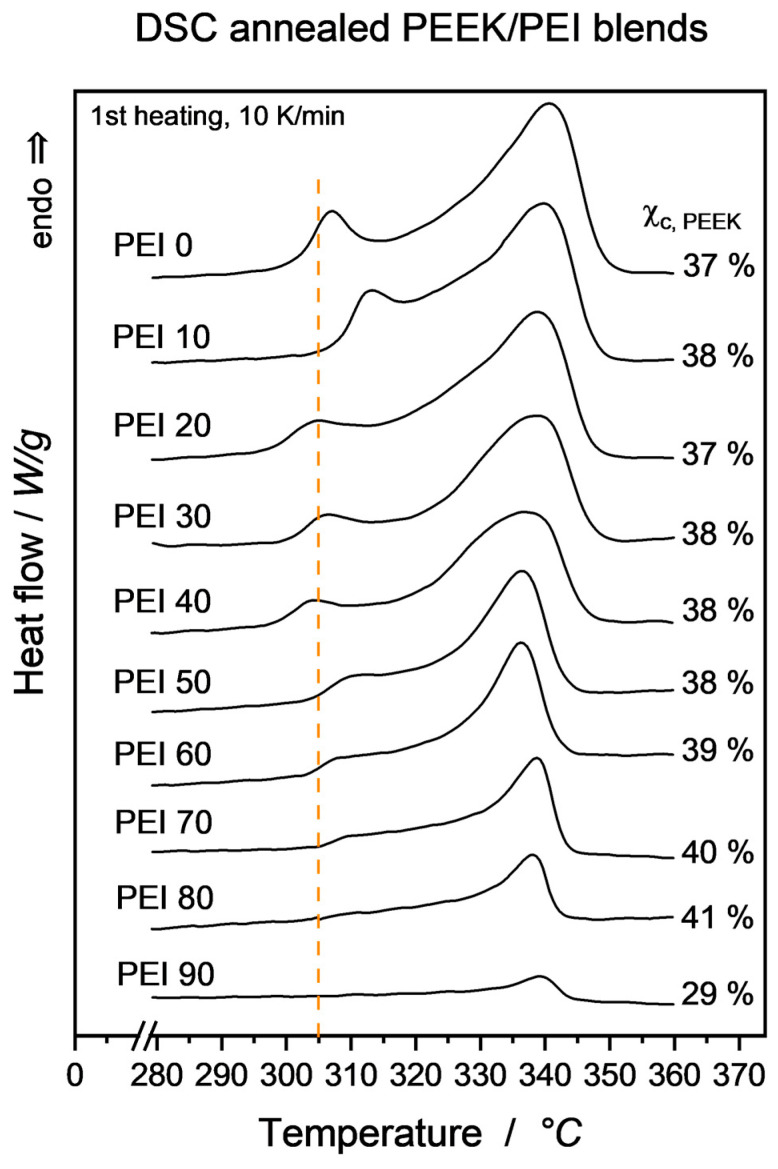
DSC curves of annealed PEEK/PEI blends. Vertical line is drawn at 305 °C annealing temperature for reference. Curves are vertically offset in the same scaling to ease comparability.

Looking at the as-processed samples, permittivity values do not show a clear correlation in regard to the blend composition. DSC analysis determined the Χ_c,PEEK_ values to scatter widely in the range of 8–16%, as can be taken from Table 3. This is due to the decreasing crystallization speeds in the PEEK/PEI system, where increasing PEI presence hinders the movement and re-alignment of PEEK molecules. This effect has extensively been investigated in the literature [19,21,50,51] and is driven i.a. by the T_g_ shift in amorphous phase to higher temperatures, corresponding with an increase in viscosity that reduces chain mobility and the resulting growth rate. To drive material crystallinity to its maximal entropically favorable state, annealing was carried out as described above and led to a much narrower scattering of Χ_c,PEEK_ in the range of PEI0–PEI80 (37–41%), even narrower in the range of PEI0–PEI60 (37–39%). The 1st heating DSC curves of the annealed samples are shown in Figure 5. It is apparent that the annealing-induced second melting peak was higher with the PEI10 blend than with all other compositions. A conclusive explanation for this cannot be provided as of now. However, this small content of PEI might act as an internal plasticizer, allowing for a higher PEEK molecule mobility during recrystallization and thus recombination of larger, higher-melting crystalline structures. Proof of this, e.g., via X-ray diffraction (XRD), is pending.

**Figure 6:**
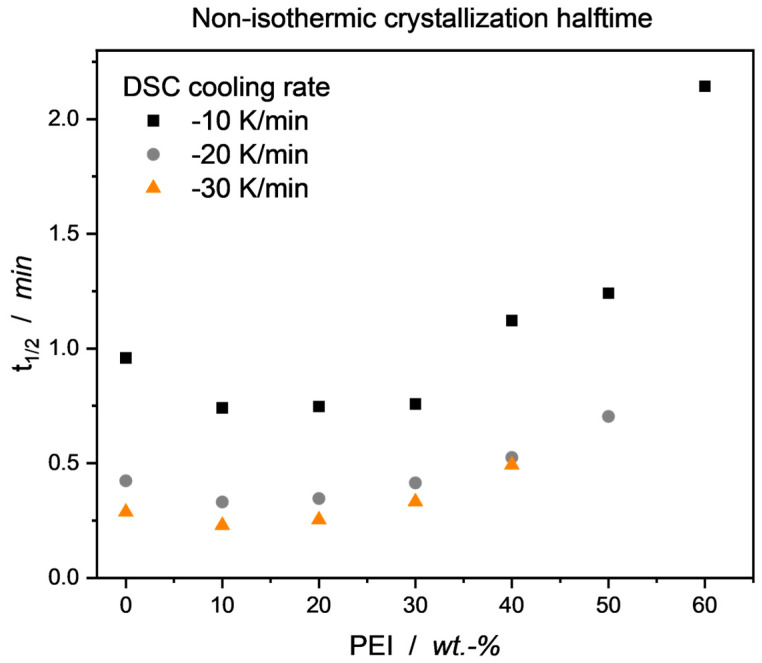
Non-isothermic crystallization halftimes for PEEK/PEI blends. In certain blend ranges, crystallization is slowed down to such an extent that no crystallization can be observed, and thus, no halftimes can be calculated (PEI70/PEI60/PEI50 for cooling rates −10/−20/−30 K/min, respectively).

As mentioned and referenced above, introducing an amorphous blend partner hinders the crystallization processes of the semicrystalline material. Figure 6 shows the blends’ crystallization halftimes for different constant DSC cooling rates. Compared to the neat PEEK, a slight drop in halftime can be observed for small PEI amounts in the blend. This is due to the PEI acting as a nucleating agent in terms of heterogeneous nucleation, while still being present in such a small amount that it does not significantly hinder the crystallization kinetics of PEEK. As expected, the faster the cooling rate, the lower the PEI content at which no crystallization can be detected anymore. For practical applications, such as injection molding with rather high cooling rates to aid a short processing cycle time, this has to be taken into account.

**Figure 7:**
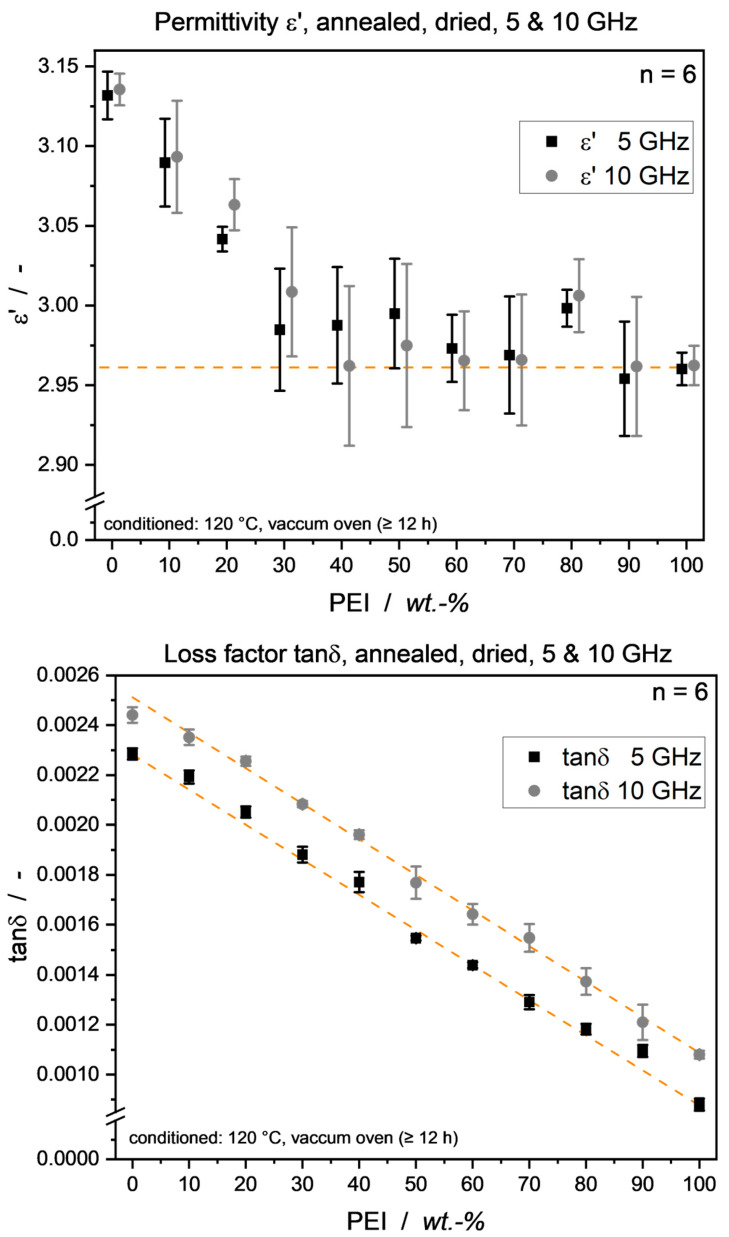
PEEK/PEI blend dielectric properties of annealed samples at 5 and 10 GHz. (**Top**): Permittivity values decreasing close to PEI level from 40 wt.-% PEI content. Note the x-axes offset only for measurement frequency distinction; blend ratio is in whole 10 wt.-% increments. Line at PEI permittivity level to guide the eye. (**Bottom**): Loss factor showing linear mixing behavior over the entire blend range. Lines show linear fit with corrected R^2^ values of 0.997 for both frequencies.

With PEEK crystallinity equalized over the blend range, a clear *ε*′ and tan*δ* behavior can be observed. As shown in Figure 7 (top), blend permittivity decreases to a plateau value at around 30–40 wt.-% PEI, intriguingly resembling that of pure PEI. As blend composition is controlled very deliberately, this is highly unlikely to be due to errors in manual blend mixing ratios and rather due to morphological effects discussed below. Effectively, blend ratio quality can be validated to be within small error margins, as Figure 7 (bottom) shows a linear mixing behavior of tan*δ* over the whole blend range. A shift to higher loss factors with increasing measurement frequency was expected and is in accordance with the usual low-loss material behavior in the GHz regime [52,53].

**Figure 8:**
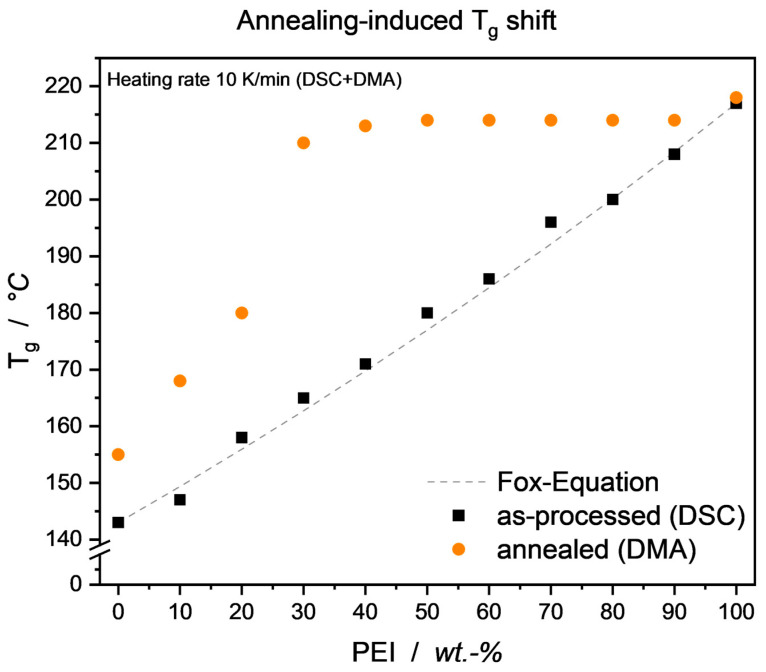
T_g_ of as-processed and annealed PEEK/PEI blends. Note that different analysis techniques DSC and DMA were employed.

Despite the rather broad scattering of permittivity values per blend ratio, the plateau effect is striking and prevailing in both measurement frequencies. As mentioned, PEEK and PEI have been proven to be miscible over the entire blend range, yet the annealing process might have led to some degree of phase segregation, nevertheless. As a matter of fact, this annealing-induced change in phase morphology of PEEK/PEI blends has been shown in the literature in the past [36,37]. An established tool to detect separated phases in thermoplastic blends is the investigation of the blend’s T_g_, with miscible blends showing a single glass transition temperature, whereas immiscible ones distinctively show multiple glass transition temperatures for every phase excluded from the homogenous mixture. T_g_ for all as-processed blend samples could clearly be read out from the DSC curve and showed a T_g_ propagation fitting well to the established Fox Equation for miscible thermoplastic blends,

(5) $$\frac{1}{T_{g}}=\frac{w_{1}}{T_{g1}}+\frac{w_{2}}{T_{g2}}$$

with the overall blend glass transition temperature T_g_ and *w*_1_, *w*_2_, T_g1_ and T_g2_ the weight fractions and glass transition temperatures of the neat blend partners, respectively. For the annealed samples, DSC measurements showed ambiguous curves not suitable to positively read out single or multiple T_g_, so DMA analysis was employed. Results are shown in Figure 8, where the heating rate was kept constant in order to allow for some degree of testing consistency. The authors are aware that due to the static vs. dynamic load environments, DMA delivers slightly higher T_g_ values compared to DSC analysis. The annealing-induced rise in neat PEEK T_g_ is in good accordance with the literature and is due to the reduced mobility of the amorphous phase. Beyond that, it can be seen that, through annealing, already at 30–40 wt.-% PEI, a blend T_g_ close to that of neat PEI is emerging.

**Figure 9:**
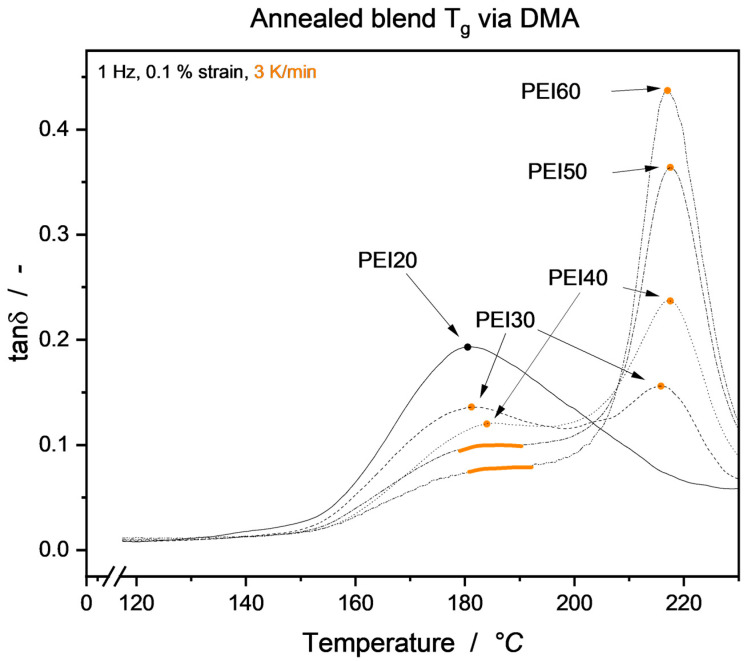
DMA loss tangent for blend T_g_ determination. A heating rate of 3 K/min reveals a double-peak behavior above 20 wt.-% PEI (highlighted). For PEI50 and PEI60, the lower temperature peak of PEEK T_g_ is masked due to its lower fraction in the blend, leading to a shoulder in the loss tangent curve.

This behavior is in good accordance with the literature and results from the realignment of PEEK molecules, that have been miscible bound with the PEI fraction, into the already formed crystalline structure, effectively reducing the overall PEEK molecular mobility in the blend. Also, excluding PEI entirely from the PEEK crystalline regions leads to an amorphous blend phase dominated to a larger extent by PEI and its thermal properties, i.e., glass transition temperature. To prove the presence of a phase segregation, samples were subjected to a slower DMA heating ramp of 3 K/min. As can be seen in Figure 9, a double-peak behavior was forming, leading to the assumption that indeed a PEI-dominated amorphous region and a PEEK-dominated crystalline region were present.

In respect to the determined permittivity values over the blend range, this separated two-phase view does not seem to be sufficient to explain the plateau at the PEI level. In this case, a volumetric mixing of permittivity values would occur, leading to a behavior similar to the observed tan*δ* mixing. Instead, it can rather be attributed to the formation of a rigid amorphous fraction (RAF) in the blend system through the annealing process. Said phase has been described in the literature for PEEK and PEEK/PEI blends [36,54,55,56]; however, its influence on material permittivity has not been investigated yet. The authors propose that this RAF combines two structurally derived properties that aid in lowering the overall material permittivity: On the one hand, the RAF being amorphous in nature offers a lower density than its crystalline counterpart. As established in 3.1, higher free volume in a bulk material leads to lower permittivity values; hence, amorphous PEEK leads to lower *ε*′ values. However, Χ_c,PEEK_ values for the samples were measured to be on similar levels of 37–41%. Determining this via DSC, the RAF was not taken into account here, as only crystalline structures were considered in the melting process. Changes in the specific heat increment ΔC_P_ at T_g_ can be used to calculate the RAF, as has been shown in [56]; the DSC measurements in this investigation, however, delivered curves not suitable for consistent ΔC_P_ calculation at T_g_. On the other hand, the “rigid” aspect seems to be much more decisive to the permittivity-reducing capabilities of the RAF. Being structurally attached to the crystalline phase, essentially immobilizing the molecular structure, this structure reduces polarizability and thus the bulk material permittivity. The presence of a RAF in the tempered samples is supported by an increase in T_g_ for the pure PEEK material from 143 °C (as-processed) to 155 °C (tempered), even increasing with the gradual addition of PEI in the material system. Hence, for the tempered blends, four structures should be considered: (I) separated amorphous PEI (no changes in mobility here compared to neat PEI), (II) a certain fraction of still-mixed amorphous PEEK and PEI, (III) the separated PEEK rigid amorphous fraction, and (IV) the PEEK crystalline phase, which is also less mobile yet more dense than amorphous PEEK. This allows for overall reduced dielectric properties in the tempered PEEK/PEI blend system, compared to each individual pure material.

As can be seen, through annealing, the pure PEEK T_g_ is increased compared to the untreated material. This leads to the changes in dielectric properties laid out above, which are caused by a higher density through re-crystallization (higher ε′) as well as reduced molecular mobility (lower tanδ), also represented through the T_g_ rise.

**Figure 10:**
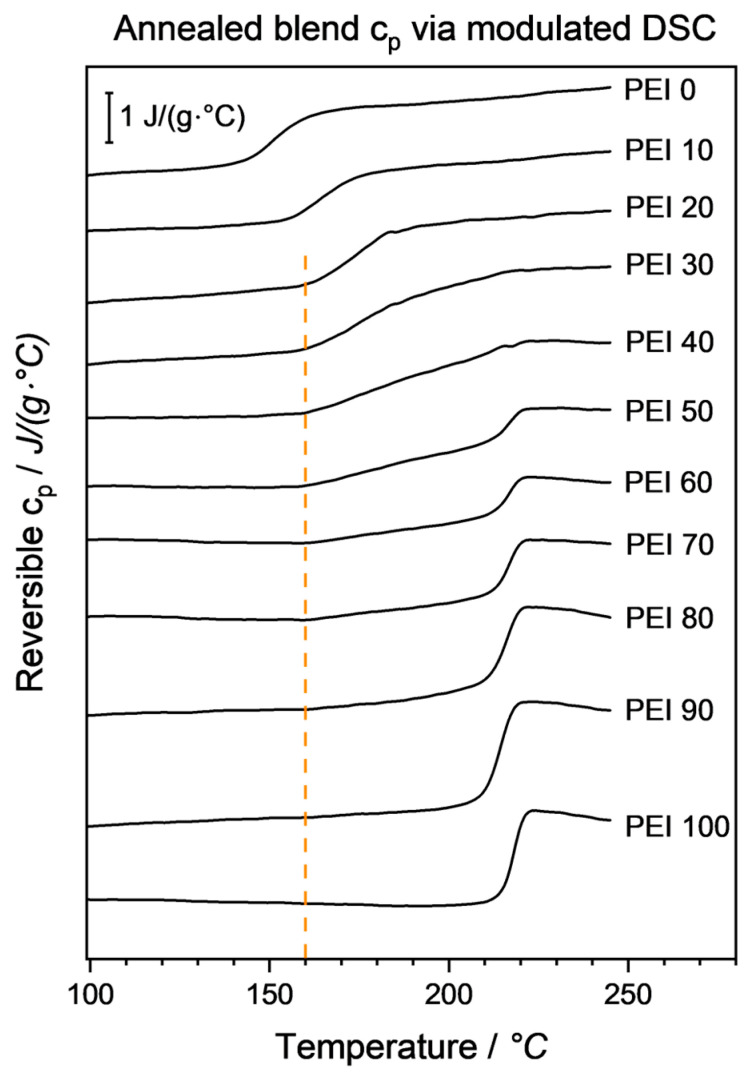
Reversible c_p_ signal for all blend ratios acquired via modulated DSC. Three general behaviors can be identified: a sharp, single T_g_ step (PEI0–PEI20); a change in c_p_ slope at ca. 163 °C followed by a sharp, single T_g_ step (PEI50–PEI80); a continuously stretched change in c_p_ slope between ca. 163 °C and ca. 217 °C (PEI T_g_). Line indicates cross-blend similar c_p_ onset. Curves are vertically offset in the same scaling to ease comparability.

With the results shown in Figure 10 as well as Table 4, in the annealed blends, three structural characteristics can be derived based on the results of the complementary measurement methods DMA and mDSC:

(I) PEI-integrated amorphous phase (10–20 wt.-% PEI): Here, the amount of PEI is completely miscible in the amorphous PEEK fraction. We observe a single T_g_ both in DMA and mDSC.

(II) PEI-excluded amorphous phase (50–90 wt.-% PEI): We see a sharp, distinctive step in the c_p_ signal at around 217 °C, representing the pure PEI T_g_. Reading the Δc_p_ at this temperature, the relative PEI exclusion from the amorphous phase can be calculated using formula (6) below, where Χ_PEI,ex_ represents the percentage of PEI excluded from the amorphous blend phase, Δc_p_ the measured change in specific heat capacity at PEI T_g_, Δc_p,PEI100_ represents the measured Δc_p_ value for pure PEI, and w_PEI_ the PEI weight-fraction in the PEEK/PEI blend system.

(6) $${Χ}_{PEI,ex}={\Delta c}_{p}^{\;}/\left({\Delta c}_{p,PEI100}^{\;}\times w_{PEI}\right)$$

As can be seen in Table 4, this exclusion affects most of the PEI fraction in the blend system, leading to calculated exclusion values of 85 to 100% (the PEI90 value only being mathematically > 100%). The small remainder of the PEI has still to be mixed in the amorphous PEEK fraction.

(III) Intermediate phase (30–40 wt.-% PEI): It can be reasoned that here, a partial yet continuous PEI segregation occurs. While DMA shows some signs of phase separation (Figure 9), mDSC does not yield two distinctive T_g_ steps. Through the initiated PEI segregation, amorphous areas with varying PEEK/PEI ratios are emerging. Since still miscible, each area renders a slightly different T_g,_ leading to a broad, continuously stretched glass transition “step” between the annealed PEEK and PEI T_g_, respectively.

All blend ratios from PEI20 onward have in common a fixed onset temperature at 162–164 °C. The visible change in c_p_ slope indicates some secondary relaxation processes happening in the PEEK-rich amorphous phase. Since most of the PEI is excluded here, and PEEK crystallinity has been measured at 38–41%, it is mostly made up from the amorphous PEEK fraction, with only a small PEI remainder in the mix. Since no relaxation processes can be observed below that temperature, such as regular mobile amorphous PEEK at around 143 °C, it can be deduced that virtually the entire amorphous PEEK has been transferred to the RAF.

In Figure 7, a distinctive increase in permittivity at 80 wt.-% PEI in the blend can be seen. The sole argument above, attributing free volume/density to permittivity readings, does fall short, as an increase in specimen density would be expected here. Table 3, however, does not show an analogous increase in density. Instead, effects of the crystalline structure have to be considered for permittivity influence. Notably, it has been shown that in a similar PEEK/PEI blend system, at 80 wt.-% PEI, the effect of interspherulitic segregation occurs, giving rise to isolated lamellae at the border of spherulites formed [57]. Incorporating this finding into the results presented here, it can be reasoned that those isolated lamellar structures show higher molecular mobility compared to those tightly bound in their respective spherulitic structures. At 90 wt.-% PEI, it can be seen that PEEK only plays a subordinate role in the blend and does not show regular crystallization behavior (Χ_c_ 29%). Over the broad remainder of the blend range, the PEEK crystalline structure emerging in the blend can be seen as independent from the PEI content, as has been proposed time and again, with the kinetics only being slowed down by the presence of amorphous PEI [19,20,57]. However, the crystalline structures emerging from annealing, coming from the glassy state of quenched samples, might have formed differently. If they were to give up their spherulitic structure above 30–40 wt.-% through that processing route, the influence of the underlying crystalline structure on the dielectric properties could be derived. This correlation, however, has still to be shown.

**Figure 11:**
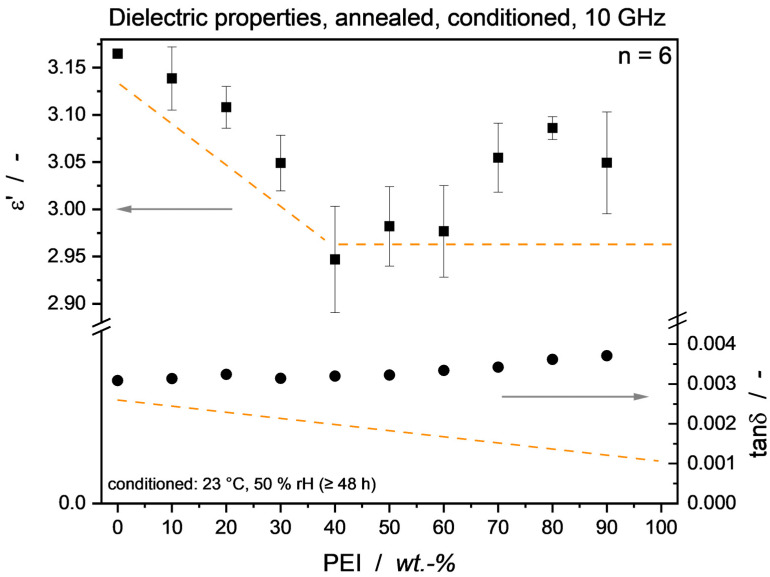
Dielectric properties of annealed PEEK/PEI material, conditioned under standard atmosphere. The influence of moisture can be seen by an increase in ε′ and tanδ values compared to the dry results. Through the higher water uptake of PEI rich blends, the loss factor is equalized over the blend range. Despite moisture presence, permittivity values show a minimum at 40 wt.-% PEI. Dashed lines indicate the dried material values as optical reference (see Figure 6).

For actual applications of PEEK/PEI blends in electronic parts, moisture influence has to be re-considered, as probably no use case involves a zero-humidity environment. To examine this, the annealed samples were conditioned under standard atmosphere, and the dielectric properties were analyzed. Figure 11 shows 10 GHz *ε*’ and tan*δ* values, revealing that, intriguingly, an *ε*’ minimum can be achieved with 40 wt.-% PEI. This is due to the permittivity-reducing effect of phase segregation laid out above, overlaid by a general increase in dielectric properties through the presence of water in the material system. The data point of 40 wt.-% PEI being lower than the dried one can be attributed to data scattering.

### 3.3. LDS-Compound Blend Ratio Identification

**Figure 12:**
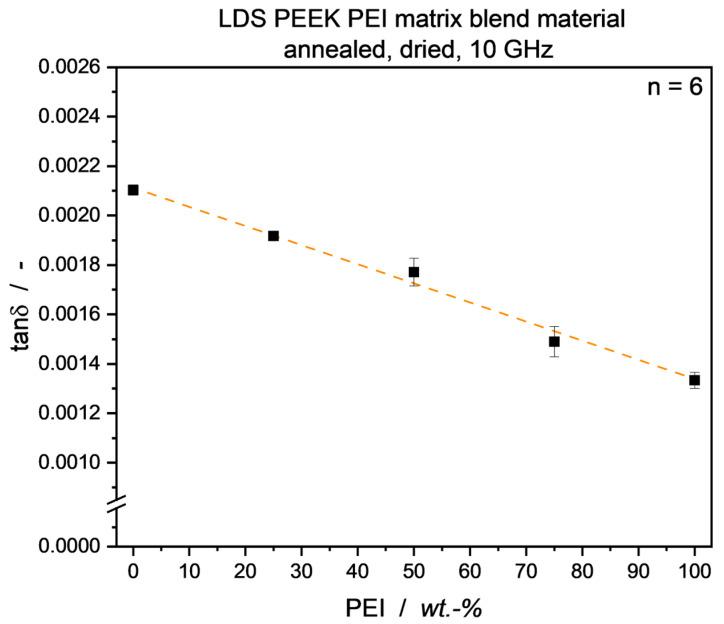
Loss factor of compounds from commercial filled PEEK and PEI material used for laser direct structuring. A linear mixing behavior similar to that of the unfilled blend system can be observed, as shown by linear interpolation with a corrected R^2^ value of 0.996.

As shown above, a linear mixing behavior concerning tan*δ* can be applied to the unfilled blend system of PEEK and PEI. In regard to possible recycling routes for thermoplastic PCB substrate materials based on the developed LDS-PEEK/PEI compounds, it is crucial to know about the composition of raw materials to be fed into recycling processes. Without going into detail here, inorganic filler materials for further mechanical stiffening and matching of the compound’s thermal expansion to that of metal conductive tracks bonded to the substrate surface are required in PCB applications. While the filler content can easily be determined e.g., via thermo-gravimetric analysis, matrix blend ratio identification of an unknown material is rather cumbersome. To prove the linear tan*δ* mixing behavior of PEEK/PEI blends even with the presence of filler material, measurements were conducted with blends of a commercial PEEK compound and a second, PEI-based compound with the same filler material. Utilizing the same processing and annealing routine as for the unfilled blends, tan*δ* values for the dried material indeed showed a linear mixing behavior, as seen in Figure 12. Given the knowledge (or measurement) of loss factors of single-material matrix compounds, this potentially provides an additional toolset for determination of matrix blend ratios in unknown blend and compound systems. Further investigation of filler/matrix interactions and the respective impact on dielectric properties still has to be thoroughly carried out. Taking the RAF for unfilled systems into consideration, the interplay with filler materials will have an additional, major impact in terms of matrix-filler-interface formation [58]. This phenomenon is beyond the scope of the presented research and will be looked into in future investigations.

## 4. Conclusions and Outlook

Investigations of dielectric properties—permittivity *ε*’ and loss factor tan*δ*—were carried out for a PEEK/PEI blend system over the whole blend range at frequencies of 5 and 10 GHz. A correlation of said properties and the material crystallinity Χ_c_ was found, showing that higher crystallinity leads to an increase in *ε*’ due to the reduction in free volume in the bulk material, and a decrease in tan*δ*, attributed to a reduction in molecular mobility. To set PEEK Χ_c_ to a fixed value in the blend system, effectively taking this variable out of consideration, samples were annealed under elevated temperatures. A permittivity reduction could be observed with increasing PEI content up to 40 wt.-%, staying close to the PEI *ε*′ plateau level for higher PEI contents. The blend loss factor followed a linear mixing rule over the whole blend range, provided the absence of moisture and a constant degree of crystallinity of the semicrystalline blend partner.

From the results presented, findings for permittivity and loss factor dependency could be derived. tan*δ* appears to be solely material-specific, i.e., depending on the mixing ratio in the blend, hinting that the loss factor of non-filled blends is based on the polymer constitution. On the other hand, *ε*′ is much more prone to changes in polymer conformation and crystallinity, as well as possible phase segregations that can be evoked through annealing processes. Additionally, the effect of an emerging rigid amorphous fraction (RAF) has to be taken into account.

In a transfer trial, the mixing rule for dielectric losses was positively shown to be applicable to a filled PEEK/PEI compound to be utilized in the laser direct structuring (LDS) process. This opens possibilities for further tools of unknown blend analysis, crucial for thermoplastic recycling processes.

To continue the research in this field and to further fill gaps in the literature, dielectric properties of other thermoplastic blends, miscible and immiscible alike, have to be analyzed at elevated frequencies in the GHz range. Here, different combinations of amorphous and semicrystalline thermoplastics will lead to insights into structure–property-relationships concerning polymer morphology and its impact on dielectric properties. Furthermore, several aspects additionally have to be investigated if a PEEK/PEI blend material is to be used in a practical application. This concerns permittivity and loss factor measurements covering the full HF spectrum required for 5G and mmWave technologies. As SPDR measurements are limited in frequency, different resonator-type setups might prove useful here. Additionally, long-term material behavior and performance have to be characterized. These affect heating/cooling cycles at varying rates, as well as performance stability under different humidity conditions. Special attention must be paid to whether the annealing-induced structures described here also survive MID soldering processes. The focus of any research should hereby be on the PEEK-rich blend ratios, as they promise the best trade-off concerning annealing-induced permittivity reduction, low moisture uptake, as well as a potentially higher heat deflection temperature compared to PEI-rich variants.

## Figures and Tables

**Table 1:** Overview of commercially available PCB substrate materials, intended for HF applications. Included are all available grades per manufacturer with permittivity values of 3.00 or below, to the best effort of the authors. Manufacturers: Rogers Corporation (Chandler, AZ, USA); AGC Inc. (Tokyo, Japan); Doosan Group (Seoul, Republic of Korea); Ventec International Group (Suzhou, China).

Manufacturer	Substrate Material Bases	*ε*′ Range/-	tan*δ* Range/-
Rogers	PTFE	2.17–3.00	0.0009–0.0021
	Resin	2.55	0.0026
AGC/Taconic	PTFE	2.17–3.00	0.0009–0.0026
	Resin	3.00	0.0025–0.0029
Doosan	PTFE	3.00	0.0013
	Resin	2.93	0.0004
Ventec	PTFE	2.94–3.00	0.0009–0.0011

**Table 2:** ε′ and tanδ values in the literature for PEEK and PEI, respectively. Publications investigating the influence of crystallinity on dielectric properties in the GHz range are not available, while published work in those frequencies does not take into account the degree of PEEK crystallization. PEI values are scattered throughout the literature. For most PEEK materials, degree of crystallinity is not explicitly given, as are tan*δ* values (annotated with n/a).

Material	Χ_c_/%	*ε*′/-	tan*δ*/-	Frequency Range/GHz	
PEEK	n/a	3.10	0.0040	1 × 10^−6^ /1 × 10^−3^	[11]
	n/a	3.18	0.0031	10	[12]
	n/a	3.31	0.0041	X-Band (8.2–12.4)	[13]
	0.6–38.8	3.52–3.78	n/a	1 × 10^−9^	[14]
PEI	-	2.51	n/a	1.0–13.5	[15]
	-	2.89	n/a	1 × 10^−3^	[16]
	-	2.27	0.0121	17	[17]

**Table 3:** Crystallinity, density and dielectric properties of annealed PEEK/PEI blend materials. As-processed crystallinity is also given for comparison. Completely amorphous PEI100 shows no crystallinity (Χ_c,PEEK_ not applicable), as does as-processed PEI90, where crystallization in hot pressing is too slow to allow for any crystallization.

Blend Composition	Χ_c,PEEK_/% As-Processed	Χ_c,PEEK_/% Annealed	Density/g/cm^3^ Annealed	*ε*′/- Annealed/Dried		tan*δ*/- Annealed/Dried	
				5 GHz	10 GHz	5 GHz	10 GHz
PEI0	14	37	1.302	3.13	3.14	0.0023	0.0024
PEI10	14	38	1.296	3.09	3.09	0.0022	0.0024
PEI20	10	37	1.285	3.04	3.06	0.0021	0.0023
PEI30	12	38	1.293	2.98	3.01	0.0019	0.0021
PEI40	16	38	1.293	2.99	2.96	0.0018	0.0020
PEI50	8	38	1.289	2.99	2.97	0.0016	0.0018
PEI60	8	39	1.284	2.97	2.97	0.0014	0.0016
PEI70	7	40	1.278	2.97	2.97	0.0013	0.0016
PEI80	11	41	1.279	3.00	3.01	0.0012	0.0014
PEI90	0	29	1.275	2.95	2.96	0.0011	0.0012
PEI100	n/a	n/a	1.269	2.96	2.96	0.0009	0.0011

**Table 4:** Calculated annealing-induced PEI exclusion across blend ranges. Note that the calculated relative PEI exclusion for PEI90 is only mathematically above 100%. For PEI90 and PEI100, no c_p_ onset temperature can be detected. PEI0 through PEI40 show no distinctive PEI glass transition step where Δc_p_ could be applied.

Blend Composition	c_p_ Onset/°C	Δc_p_/J/(g °C) at PEI T_g_	Relative PEI Exclusion
PEI0	144	n/a	n/a
PEI10	156	n/a	n/a
PEI20	163	n/a	n/a
PEI30	164	n/a	n/a
PEI40	162	n/a	n/a
PEI50	162	0.0791	93.2%
PEI60	162	0.0865	84.9%
PEI70	164	0.1102	92.7%
PEI80	164	0.1353	99.6%
PEI90	n/a	0.1607	105.1%
PEI100	n/a	0.1698	100.0%

## Data Availability

The raw data supporting the conclusions of this article will be made available by the authors on request. Further inquiries can be directed to the corresponding author.
